# Tracking of stem cells *in vivo* for cardiovascular applications

**DOI:** 10.1186/1532-429X-16-7

**Published:** 2014-01-10

**Authors:** Nicole Azene, Yingli Fu, Jeremy Maurer, Dara L Kraitchman

**Affiliations:** 1 Russell H. Morgan Department of Radiology and Radiological Science, The Johns Hopkins University, Baltimore, MD, USA; 2 Department of Molecular and Comparative Pathobiology, The Johns Hopkins University, Baltimore, MD, USA; 3 Russell H. Morgan Department of Radiology and Radiological Science, The Johns Hopkins University School of Medicine, 600 N. Wolfe Street, 314 Park Building, Baltimore, MD 21287, USA

**Keywords:** Cell tracking, Cell labeling, Stem cells, Cardiovascular disease, Computed tomography, Fluoroscopy, Cardiovascular magnetic resonance, Radionuclide imaging, Optical imaging, Ultrasound, Image-guided therapy

## Abstract

In the past ten years, the concept of injecting stem and progenitor cells to assist with rebuilding damaged blood vessels and myocardial tissue after injury in the heart and peripheral vasculature has moved from bench to bedside. Non-invasive imaging can not only provide a means to assess cardiac repair and, thereby, cellular therapy efficacy but also a means to confirm cell delivery and engraftment after administration. In this first of a two-part review, we will review the different types of cellular labeling techniques and the application of these techniques in cardiovascular magnetic resonance and ultrasound. In addition, we provide a synopsis of the cardiac cellular clinical trials that have been performed to-date.

## Introduction

**Figure 1:**
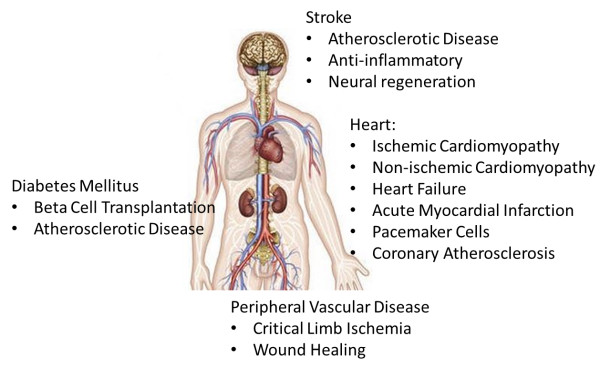
**Stem cell therapies can be envisioned to treat a wide variety of cardiovascular diseases ranging from preventing adverse remodeling in ischemic and non**-**ischemic heart disease, the creation of new pacemaker cells, replacement of beta cells in Diabetes Mellitus, and mitigating atherosclerotic disease leading to peripheral vascular disease as well as stroke.**

In 2008, an estimated 17.3 million deaths were attributable worldwide to cardiovascular diseases^a^ making cardiovascular disease the leading cause of death worldwide. Coronary heart disease (CHD) or diseases of the blood vessels supplying the heart represents approximately 42% of these deaths.^a^ Because the heart lacks any significant regenerative capacity, patients who do not die acutely after myocardial infarction (MI) are at risk for development of heart failure (HF) and sudden cardiac death related to ventricular arrhythmias. Among other factors, the extent of myocardial damage is largely predictive of the likelihood of future cardiac dysfunction and HF. Stem cell therapy holds the promise of salvaging or reconstituting the electromechanical function of damaged heart tissue; thereby preventing, mitigating, or possibly reversing CHD-related heart failure and ventricular arrhythmia. Moreover, there are a wide range of additional possible therapeutic uses of stem cell to treat cardiovascular diseases (Figure 1).

Stem cells have two main characteristics: 1) the ability to undergo clonal replication and 2) the capacity to differentiate into multiple cell types. Pluripotent stem cells can become any cell type in the body with embryonic stem cells as a classic example. Further, adult somatic cells have recently been re-programmed to take on the characteristics of embryonic stem cells —the so-called induced pluripotent stem cells (iPS). Adult stem cells (or stem cells derived from adult or non-embryonic/fetal tissue) are generally considered to be multipotent, as opposed to pluripotent, because of their inability to differentiate into all lineages of cells. Due to the ease at which adult stem and progenitor cells can be obtained and the lack of ethical issues, these stem cells have been the most extensively studied for cardiovascular applications. Examples of adult stem cells include bone marrow- and adipose-derived mesenchymal stem cells (MSC), bone marrow mononuclear stem cells, endothelial progenitor cells (EPC), and cardiac-derived stem cells/cardiospheres (CSCs) [1,2].

**Figure 2:**
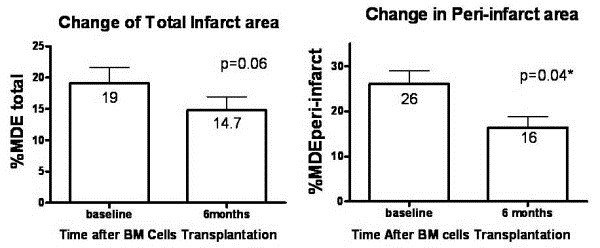
**Treatment effect of bone marrow cells (BM) implantation on percentage of total infarct area and peri**-**infarct area in the BM group as determined by CMR.** Data presented as mean ± SD (error bar). Reprinted with permission from Chan *et al.*[11].

Numerous patient studies using stem cell therapy for CHD have already been performed (Table 1). Frequently, major adverse cardiac events (MACE) are used as an end point, such as repeat myocardial infarction or vascular procedures or death. Alternatively, imaging as a surrogate endpoint for MACE can provide accurate and reproducible measures of cardiac structure and function [3]. Furthermore, imaging can provide a quantitative measure for characterizing and comparing the efficacy of stem cell therapies in the treatment of acute myocardial infarction and heart failure. In turn, imaging may provide the ability to infer the fate of the stem cells whereas MACE endpoints cannot. With imaging as a primary outcome, mixed results have been reported for clinical trials of stem cell therapy in cardiovascular disease [4-11]. One study reported greater improved left ventricular (LV) ejection fraction in the stem cell treatment group versus controls (TOPCARE-AMI) [4,5]. A second study found initial improvements but no sustained difference in LV ejection fraction, LV end-diastolic volume, and LV end-systolic volume between groups (BOOST) [7]. A third study reported no improvement in LVEF in the control group compared to the stem cell group (ASTAMI) [8]. Whereas, a fourth study using transendocardial administration of stem cells resulted in significant positive remodeling of the peri-infarction region (PROTECT-CAD) (Figure 2) [10,11]. A fifth representative study using cardiac-derived stem cells showed greater increases in left ventricular mass and larger decreases in infarcted myocardium in the cell-treated patients [9]. The combination of these clinical studies, especially those with imaging data, and meta-analyses of these trials, [12-16] suggests that stem cell administration is safe, can provide improvements in cardiac function when stem cells are administered at the appropriate time, improvements in cardiac function appear to be related more to paracrine than direct cell incorporation, some routes of administration may provide more favorable results, and timing of cell delivery may be critical depending on the type of cardiovascular disease.

**Table 1:** Clinical trials utilizing stem cells for the treatment of cardiovascular disorders

**Trial**	**Condition**	**Cell types**	**Delivery Route**	**Select Functional Results**
Strauer *et al.*[17]	AMI	BMC	Intracoronary	Increase in stroke volume index and ejection fraction. Significant decrease in ESV. Significant increase in the ratio of systolic pressure to end-systolic volume.
Kuethe *et al.*[18]	AMI	BMC	Intracoronary	No improvement of LVEF, regional wall motion at infarcted zone, contractility index, coronary blood flow reserve or maximal oxygen uptake at 3-months. No change in LV EF at 12 months.
BOOST [7,19-21]	AMI	BMC	Intracoronary	Overall treatment effect of BMC transfer on E/A. Significantly lower E/A ratio at 6 and 18 months for control group. No difference in E/A ratio at 60 months between groups. No overall effect of BMC implantation on E(a)/A(a) ratio, DT, IVRT, and E/E(a) ratio.
REPAIR-AMI [4,5]	AMI	BMC or CPCs	Intracoronary	No significant difference in LV volumes between groups, although a trend toward smaller ESVs in the BMC group; significantly improved relative infarct size and regional contractility among BMC recipients.
ASTAMI [8,22]	STEMI	BMC	Intracoronary	No significant differences between groups in change of global LV systolic function at 3 years. Larger improvement in exercise time from 2–3 weeks to 3 years in BMC recipients, but no difference in peak oxygen consumption.
REGENT [23]	AMI	Selected (CD34 + CXCR+) BMC, unselected BMC	Intracoronary	Increased LV EF at 6 months in unselected and selected BMC recipients, but unchanged for control group. No significant differences in absolute changes of LV EF between groups. No significant differences in absolute changes of LV ESV and LV EDV for all groups.
TECAM [24]	STEMI	BMC	Intracoronary	At 9 months, no significant changes in changes in minimum lumen diameter and the percentage of stenosis at follow-up between BMC and control group; no significant changes in the contralateral artery; and no changes in maximum area stenosis and plaque volume.
Hopp *et al.* (subgroup of ASTAMI) [25]	STEMI	BMC	Intracoronary	For controls, improved global and regional LV function at 6 months versus 2–3 weeks; significantly more than in the BMC group. Significant decrease in LV infarct mass; significantly more pronounced than the BMC group.
SWISS-AMI [26]	AMI	BMC	Intracoronary	Intracoronary BMMC did not improve LV function by CMR at 4 months relative to controls whether infused at 5–7 days or 3–4 weeks. Early reperfusion (<4.5 h) after STEMI predictive of more benefit from BMMC.
TIME [27,28]	AMI	BMC	Intracoronary	STEMI patients treated with PCI treated with intracoronary administration of autologous BMCs did not show improved left ventricular function at 6 months or 1 year whether treated at 3 or 7 days after PCI.
LateTIME [6]	AMI	BMC	Intracoronary	Delayed (2–3 weeks) intracoronary injection of BMCs does not improve LVEF or regional wall motion or decrease infarct size based on CMR compared to placebo-treated patients.
Fernandez-Aviles *et al.*[29]	CMI	BMC	Intracoronary	At 6 months among BMC recipients there was decreased ESV, improvement of regional and global LV function, and increased thickness of the infarcted wall. No changes in control group.
IACT [30]	CMI	BMC	Intracoronary	At 3 months post BMC administration: decreased myocardial infarct size; improved global and regional LV function; improved maximum oxygen uptake; and improved regional myocardial metabolism relative to non-treated controls.
Brehm *et al. *[31]	CMI	BMC	Intracoronary	Reduced infarct size, increased global LV EF and infarction wall-movement velocity for BMC recipients; no significant changes for control group. Improved maximum oxygen uptake increased regional (18)F-FDG uptake into infarcted tissue.
Janssens *et al.*[32]	CMI	BMC	Intracoronary	Increased mean global LVEF at 4 months in controls and BMC recipients; Decreased myocardial infarct size and better recovery of regional systolic function in BMC group; Increased myocardial perfusion and metabolism in controls and BMC patients.
Galinanes *et al.*[33]	CMI	BMC	Intramyocardial	Unmanipulated BMCs improved global and regional LV function at 6 weeks and 10 months for BMC that received CABG.
Fuchs *et al.*[34,35]	CMI	BMC	Transendocardial	Among BMC recipients, stable ED LV volume; significant improvementof ESV and EF; improved regional contractility. No significant improvements among controls.
Perin *et al.*[36,37]	CMI	BMC	Transendocardial	Improved LV EF from baseline and reduction in EDV in treated patients at 4 months. Significant mechanical improvement of injected segments at 4 months.
PROTECT-CAD [10,11]	CMI	BMC	Transendocardial	After 6 months, significant increase in exercise treadmill time and LV F in BMC recipients. Significant decrease in percentage area of peri-infarct regions; increase in global LVEF, percentage of regional wall thickening, and MPR over target area at 6-months.
TABMMI [38]	CMI	BMC	Transendocardial	Transmyocardial delivery is safe with trends toward improved cardiac function in a non-randomized pilot trial.
vanRamshorst *et al.*[39]	CMI	BMC	Transendocardial	Significant increase in LV EF for BMC recipients. Filling pressure estimate E/E’ ratio improved at 3 months in BMC group; no improvement in placebo group; significantly larger improvement in E/E(a) ratio for BMC recipients. Significant increase in E/A peak flow ratio in BMC group.
Focus-CCTRN [40]	CMI	BMC	Transendocardial	No improvement in cardiac function with autologous BMMC delivered transendocardially.
Silva *et al.*[41]	Heart failure	BMC	Transendocardial	Improved mVO_2_ and METs for treated patients at 2 and 6 months. No significant difference in ESV, EDV, and LV EF from baseline to 2 or 6 months.
Focus-HF [42]	Heart Failure	BMC	Transendocardial	Younger patients had improved cell function with improved responses compared to older patients.
TOPCARE-AMI [5,43-45]	AMI	CPC/BMC	Intracoronary	Persistent improvement of LV EF, significantly decreased LV ESV, and stable LV EDV through 5-year follow up. Significant reduction in functional infarct size.
TOPCARE-CHD [46]	CMI	CPC/BMC	Intracoronary	Cross-over study from TOP-CARE AMI. Significantly greater LV EF among BMC vs. CPC recipients and controls. Significant increase in global and regional LV function for BMC recipients, irrespective of cross-over status.
Bartunek *et al.*[47]	AMI	CD133 + BMC	Intracoronary	Significantincrease in LV EF and regional chordae shortening^;^ associated increase in contractilityand decrease in resting MIBI perfusion defect.
COMPARE-AMI [48]	AMI	CD133+ BMC	Intracoronary	LVEF improved at 4 months and 1 year compared to placebo treatment.
Goussetis *et al.*[49]	CMI	CD133 + BMC/CD133-CD34 + BMC	Intracoronary	Uptake of cells in the chronic ischemic myocardium.
Stamm *et al.*[50,51]	AMI	CD133+ BMC	Transendocardial	Enhanced global LV function and improved infarct tissue perfusion in 66% and 83% of BMC recipients, respectively.
Stamm *et al.*[52]	Chronic Ischemic HD	CD133+ BMC	Intramyocardial	Among CABG and cell therapy (vs. CABG alone) recipients, increased LVEF over baseline at discharge, 6, and 18 months and greater improvement in perfusion at the infarction zone.
Losordo *et al.*[53]	CMI	CD34+,G-CSF mobilized PBC	Transendocardial	Improved exercise time at 3 months in placebo and active treatment groups; slightly greater magnitude of improvement in CMI recipients.
ACTC34-CMI [54]	CMI/Refractory Angina	CD34+ cells	Transendocardial	Decreased frequency of angina and improved exercise tolerance
Choi *et al.*[55]	AMI	G-CSF mobilized PBC	Intracoronary	Significantly improved LVEF for cell therapy recipients after 6 months.
MAGIC Cell-DES [56]	AMI/CMI	G-CSF mobilized PBC	Intracoronary	Significant improvement in LVEF and ESV in cell recipients. In CMI patients, no significant change in LVEF and ventricular remodeling; although, significant improvement of coronary flow reserve.
Chachques *et al.*[57]	MI	Skeletal myoblast	Intramyocardial	serum incubation during cell culture reduces immunological rejection of myoblasts. Significantly improved LV EF and regional wall motion score index in cell-treated segments.
Dib *et al.*[58,59]	MI	Skeletal myoblast	Intramyocardial	For CABG patients receiving cell transplants there was significant improvement in mean LV EF; increased tissue viability; and reduced ventricular systolic and diastolic volumes.
Herreros *et al.*[60]	MI	Skeletal myoblast	Intramyocardial	In the myoblast group, LVEF, regional contractility (in cardiac segments), global and regional viability and perfusion improved significantly by 12 months.
Gavira et. al. [61]				
Ince *et al.*[62]	MI	Skeletal myoblast	Transendocardial	Increased LVEF at 12 months and significantly improved walking distance were at 1 year for myoblast recipients.
Hagège *et al.*[63]	Heart failure	Skeletal myoblast	Intramyocardial	Increased LV EF at 1-month and remained stable thereafter (median follow up of 52 months) for myoblast recipients. ACD implantation can reduce arrhythmia risk.
Siminiak *et al.*[64]	AMI	Skeletal myoblast	Intramyocardial	Significantly increased L EF at 4 months; maintained through 12 month follow up.
POZNAN [65]	Heart failure	Skeletal myoblast	Transcoronary venous	Increased ejection fraction (3-8%) in two-thirds of cases.
Smits *et al.*[66]	MI/Heart failure	Skeletal myoblast	Transendocardial	Significantly increased LVEF at 3 months, but not at 6 months. At 3 months, significantly increased wall thickening at target areas and less wall thickening in remote areas.
MAGIC [67,68]	CMI	Skeletal myoblast	Intramyocardial	No significant improvement of regional or global LV function for cell groups; significant decrease in LV volumes in high-dose cell group vs. placebo group.
Veltman *et al. *[69]	CMI	Skeletal myoblast	Intramyocardial	No sustained improvement in 14 patients compared to matched controls at 4 year follow-up.
Chen *et al. *[70,71]	AMI	MSC	Intracoronary	Regional wall movement velocity increased significantly in the MSC group, but not controls. Significantly increased LVEF at 3 months in MSC group compared with baseline and control group. Significantly improved perfusion defect in BMSC group at 3 months compared with control group with synchronous decrease in LV EDV and ESV. Significantly increased ESP: ESV.
Chen *et al.*[72]	CMI	MSC	Intracoronary	For MSC recipients, significant decrease in defect at 12 months; significantly improved level of exercise tolerance and LVEF at 3 months.
Hare *et al.*[73]	AMI	Allogeneic MSC	Intravenous	Increased LVEF in MSC recipients in CMR subset.
MSC-HF [74]	Heart Failure	MSC	Transendocardial	Currently enrolling.
POSEIDON [75]	CMI	Autologous or Allogeneic MSC	Transendocardial	Allogeneic administration of MSCs is safe and has similar improvements as autologous.
TAC-HFT [76,77]	CMI	MSC or BMC	Transendocardial	Safety of transendocardial delivery of MSCs and BMCs in patients with CMI was found to be safe.
MyStromalCell Trial [78]	CMI	ASC	Transendocardial	Currently enrolling using adipose-derived stem cells primed with VEGF-A towards an endothelial progenitor lineage.
Frils *et al.*[79]	Refractory Angina	MSC	Transendocardial	Improved LVEF and systolic wall thickening in CMR subset.
Katritsis *et al.*[80]	AMI	EPC/MSC	Intracoronary	Significantly lower wall motion score index at 4 months in MSC group; Improved myocardial contractility in ≥ 1 previously nonviable myocardial segment and restored uptake of ^99m^Tc in ≥ 1 previously nonviable myocardial scars for BMSC recipients.
Lasala *et al.*[81]	CAD	EPC/MSC	Intracoronary	Significant improvements in LV EF and significant decrease in myocardial ischemia at 1 and 6 months.

*Abbreviations*: *AMI* acute myocardial infarction, *BMC* bone marrow cell, *E* peak early transmitral velocity, *A* peak late transmitral velocity, *E(a)* early diastolic velocity, *A(a)* late diastolic velocity, *DT* E-wave deceleration time, *IVRT* isovolumic relaxation time, *BM* bone marrow, *LV* left ventricular, *ESV* end-systolic volume, *EF* ejection fraction, *EDV* end-diastolic volume, *CAD* coronary arterial disease, *CMI* chronic myocardial infarction, *F* fluorine, *FDG* fluordeoxyglucose, *MI* myocardial infarction, *BMMC* mononuclear bone marrow cell, *STEMI* ST-elevation myocardial infarction, *mVO2* myocardial volume oxygen consumption, *MPR* myocardial perfusion reserve, *METs* metabolic equivalents, *CPC* circulating blood derived progenitor cells, *HD* heart disease, *CD133+/CD34*+ bone marrow-derived CD133+ or CD34+ cells, *G-CSF* granulocyte colony stimulating factor, *PBC* peripheral blood cell, *MI* myocardial infarction, *CABG* coronary artery bypass graft surgery, *MSC* bone marrow-derived mesenchymal stem cells, *ASC* adipose-derived stem cells, *ESP* end-systolic pressure, *Tc* Technetium.

Aside from assessing stem cell efficacy, imaging could be an invaluable tool for monitoring delivery and/or tracking stem cells fate by offering real-time guidance of stem cell transplantation, cell transit, and engraftment. In addition, the results of clinical trials could be tested in relevant preclinical models to understand the mechanisms underlying enhanced benefit from delivering stem cells at a certain time point, via certain routes, with certain cell types or in certain disease entities. The efficacy of stem cell therapy has been explored using all standard clinical imaging modalities, i.e., cardiovascular magnetic resonance (CMR), ultrasound (US), X-ray/computed tomography (CT), positron emission tomography (PET), and single-photon emission computed tomography (SPECT), along with optical imaging modalities (OI), such as bioluminescence and fluorescence, in animal CHD models. To date, no consensus, however, has been reached on the best imaging modality to determine stem cell therapy efficacy. Indeed, the “best” imaging modality may be highly dependent on the availability of imaging equipment, the suitability depending on the patient population, e.g., pediatrics vs. implanted devices, or the method of delivery, e.g., intra-operatively vs. percutaneous.

For the purpose of stem cell tracking, the choice of imaging system is integral to a cell labeling strategy; each possessing advantages and disadvantages for cardiovascular imaging, in general, and stem cell tracking, in particular (Table 2). The ideal imaging system is one that does not produce ionizing radiation but has high spatial resolution (ideally single cells), temporal resolution (for imaging the beating heart), contrast (i.e. “soft tissue” resolution), and sensitivity to a small number of cells or at a minimum a therapeutic dose. Further, it should be commonly used clinically, require minimal operator dependence, be inexpensive, and elicit minimal or no patient discomfort. Importantly, no single imaging technique for tracking stem cells has all the ideal characteristics. However, multimodality approaches can sometimes exploit the advantages of each modality while moderating their respective disadvantages.

**Table 2:** **Stem cell tracking strategies for cardiovascular applications *in vivo***

**Physical principle**		**Modality**	**Cell labels**	**Advantages**	**Disadvantages**
Tissue contrast based	Magnetic field change	CMR	• Iron oxides	• High spatial resolution	• Low sensitivity
			• Gad-chelates		• Signal not linked to cell viability
			• Microcapsules	• High anatomic detail	
			• Reporter genes (enzyme-based, transporter-based)		• Lack of CMR-compatible devices for interactivity
				• No ionizing radiation	
				• Post-processing capabilities	
					• Not compatible for patients with implants
				• Permits medium-term tracking	
					• Expensive
					• Acoustic noise
	Electron density change	X-Ray/CT	• Gold Nanoparticles	• High sensitivity	• Ionizing radiation
			• Microcapsules	• High potential of real-time interactivity	• Limited spatial resolution
					• Lacks soft tissue detail
	Echogenicity change	US	• Liposomes	• High potential of real-time interactivity	• Difficultly with thin/obese patients
			• Microbubbles		
			• Microcapsules		• Highly operator dependent
			• Perfluorocarbons	• No ionizing radiation	
					• Interpretation has high learning curve
				• Inexpensive	
				• Highly portable	
					• Limited resolution
					• Acoustic artifacts may compromise image
Photon emission based	Radionuclide imaging (High energy ionizing radiation)	PET	• Reporter genes, e.g. HSV-tk, hNIS	• High sensitivity	• Poor anatomic detail
				• High translational capacity	
					• Poor interactivity
			• Radionuclides, e.g. ^18^ F-FHBG, ^124^I FIAU, and ^18^ F-FDG		• Ionizing radiation
					• Temporal limitations (due to radioactive decay)
		SPECT	• Radionuclides, e.g. ^111^In oxine, ^99m^Tc and^18^ F FDG		• Concerns for label-induced cellular toxicity
					• Biohazardous labels
					• Expensive
	Optical imaging (Low energy radiation)	BLI	• Reporter genes, e.g. luciferase	• Permits longitudinal monitoring	• Limited spatial resolution
					• Lacks clinical relevance
				• Low background	
				• No excitation light required	• Biohazardous labels
		Fluorescence	• Fluorophores, e.g. GFP	• High sensitivity	• Photon attenuation w/cell division
				• Multiplexing	
			• Near-infrared probes	• No ionizing radiation	• Autofluorescence yields high background
			• Quantum dots	• Low cost	
					• Small depth of high-resolution
				• Permits short-term tracking	
					• Biohazardous labels

BLI: Bioluminescence imaging; ^18^F FDG: Fluoro-2-deoxy-d-glucose; FHBG: Fluoro-3-hydroxymethylbutyl; GFP: green fluorescent protein; ^111^In: Indium; PET: Positron emission tomography; SPECT: Single photoelectron computed tomography; ^99m^Tc: Technetium; US: Ultrasound.

When choosing a cellular labeling strategy, one should reflect on the characteristics of the perfect cellular label. Ideally, the cellular label:1) is biocompatible and nontoxic, 2) requires no genetic modification of stem cells, 3) involves minimal or no dilution of the label with cell division, 4) involves minimal or no transfer of the label to non-transplanted cells, 5) possesses long-term stability over months to years *in vivo*, 6) is quantifiable and proportional to the cell number, 7) does not necessitate injection of a contrast agent, 8) does not interfere with normal cell function, and 9) is inexpensive [82]. It might also be advantageous to have cell labeling schemes that identify cells that have differentiated down a specific lineage or can destroy cells that have differentiated down an unwanted pathway.

Generally, cellular labeling techniques are classified as receptor-based techniques, reporter gene labeling, or direct labeling techniques. As the stem cell undergoes differentiation, specific cell surface markers evolve thereby limiting the relevance of receptor-based labeling techniques in stem cell therapy. Further, reporter gene labeling inherently alters the stem cell’s genetic material, bringing about safety concerns likely to lengthen the time to clinical acceptance. Currently, direct labeling techniques serve as the primary means of labeling stem cells for *in vivo* cardiovascular applications. With direct labeling, the cellular marker (e.g. fluorescence probes, MR contrast agents, and radionuclides) is taken up into the cell or attaches to its surface; often direct cell labeling is performed *in vitro* prior to transplantation.

Many recent reviews describing tracking strategies for studying stem cell based cardiac therapies are available in the literature [83-97]. In this first of a two part review, we will summarize the approaches, advantages, and disadvantages of stem cell tracking strategies for cardiovascular applications and specifically highlight recent developments in this rapidly developing field (Table 1) with a particular emphasis on ultrasound and magnetic resonance imaging technology. In part two of this review, we will concentrate on optical and radionuclide imaging technologies and discuss the growing use of multimodality imaging techniques as well as our impressions regarding the future of stem cell imaging in cardiac therapy.

### Noninvasive imaging modalities for stem cell tracking

The noninvasive imaging modalities employed in stem cell tracking for cardiovascular applications *in vivo* include ultrasound, CMR, CT/X-ray fluoroscopy, radionuclide imaging, and optical imaging. As mentioned, each modality possesses its own set of advantages and disadvantages, irrespective of the cell labeling strategy employed. While anatomical localization using these imaging techniques is based on the ability to differentiate between tissue types, the intrinsic contrast of stem cells relative to native heart tissue is very low. Thus, stem cells must be labeled either before or after transplantation to detect them relative to the surrounding tissue. Methodologies to label stem cells are described in greater detail below by imaging modality, along with unique advantages and disadvantages to each labeling method.

CT/X-ray fluoroscopy, CMR, and US all depend on physical properties which impart image contrast. In each of these modalities, the final image is composed of signal intensities that are transformed into gray scale images corresponding to tissues possessing different physical properties. In CT/fluoroscopy, CMR, and US the measured physical properties are electron density, nuclear dipole relaxation time, and acoustic reflection (echogenicity), respectively. CT provides the highest spatial resolution while CMR provides the greatest soft tissue contrast. X-ray fluoroscopy and US provide higher temporal resolution relative to CMR. Using a multimodality imaging approach, such as highly interactive fluoroscopy in combination with one having greater anatomic detail (e.g., CTor CMR), may improve the accuracy of stem cell placement as well as provide confirmation of initial post-procedural targeting.

Unlike tissue-contrast based imaging, photon emission-based imaging modalities (e.g., PET, SPECT and OI) generate images by detecting the release of light or other forms of electromagnetic radiation. In PET, the radiotracer undergoes decay and emits a positron that travels in tissue subsequently encountering an electron. Each positron-electron “coincident” event results in an annihilation pair that emits two gamma ray photons in the opposite direction. Image acquisition is based on the external detection of the emitted gamma pairs. SPECT is similar to PET in its usage of a radioactive tracer and image acquisition based on detection of gamma rays. However, the radiotracer used in SPECT emits gamma radiation that is measured in two-dimensional projections that are reconstructed into a tomographic image, without a “coincident” event. This difference accounts for the higher sensitivity obtained from PET versus SPECT scans.

The OI modalities of bioluminescence and fluorescence are photon emission-based as well; whereby electrons in an excited state emit a photon upon returning to the ground state with light subsequently being emitted in a defined wavelength. The fundamental difference between bioluminescence and fluorescence is the mechanism by which the excited state is generated. Bioluminescent photoproteins, such as luceferins, emit light as a byproduct of a chemical reaction. Fluorescent compounds, also called fluorophores (e.g., green fluorescent protein or GFP or quantum dots), undergo excitation by incident light and typically emit light at a different wavelength that can be detected.

Because photon emission-based imaging techniques do not measure tissue contrast, anatomic localization can be difficult. Moreover, attenuation of photons by the tissue can further complicate imaging in deep structures. For these reasons, photon emission-based images are usually acquired and interpreted in conjunction with tissue-contrast based images; whereby the lack of tissue contrast and relatively low spatial resolution of photon imaging techniques is balanced by the exquisitely high sensitivity of the anatomical imaging technique, such as CT.

In the clinical setting, PET-CT and SPECT-CT are commonly used, and PET-MR is currently being rapidly developed for clinical applications [98]. In general, photon emission-based imaging techniques have higher sensitivity because of the introduction of non-native substances that are measured. Thus, there is typically no background signal that must be overcome for detection with the exception of tissue autofluorescence for optical imaging techniques. Photon emitting radiotracers are also very useful in imaging of metabolic processes. Using radioisotopes to label biologically important analogues and observe their behavior *in vivo* is useful both clinically and experimentally. For example, consider the lipophilic nuclear cardiac stress-test radiopharmaceutical Technetium (^99m^Tc) sestamibi. Technetium (^99m^Tc) sestamibi is distributed proportionally to myocardial blood flow and freely crosses mitochondrial membranes where it is accumulated, allowing a “snapshot” of cardiac perfusion to be obtained at rest and during cardiac stress [99]. Hence, multimodality imaging can be used to exploit advantages while mitigating the disadvantages in an individual imaging modality.

### Stem cell labeling approaches by imaging modality

#### Ultrasound

Among the noninvasive imaging modalities, US continues to be the most commonly utilized for cardiac structure and function evaluations, in part due to its non-invasiveness, low cost, and portability. Moreover, the real-time interactivity of US and lack of ionizing radiation support the expansion of this imaging modality for stem cell delivery and tracking. However, until recently, ultrasound has been scarcely utilized in stem cell tracking because of the difficulty of attaching a long-lived ultrasound-visible label.

Tracking of stem cells via ultrasound can be done in combination with microbubble contrast agents, acoustically active liposomes, or perfluorocarbon nanoparticles. As previously stated, receptor labeling of stem cells is problematic because stem cells often lose markers as they differentiate as well as the inability to target exogenously delivered stem cells from native cells. One approach to overcome this problem has been spearheaded by Leong-Poi and co-workers, who have genetically modified endothelial progenitor cells (EPCs) to express the mouse H-2Kk protein [100]. Matrigel plugs containing H-2Kk-expressing EPCs were implanted subcutaneously in rats and subsequent ultrasound imaging demonstrated *in vivo* targeting of lipid microbubbles with anti-H-2Kk antibodies to the matrigel plugs whereas non-targeted microbubbles were not visible ultrasonically [100]. Beyond the disadvantage of genetic modification of stem cells, this promising approach also will suffer from problems of delivery of the microbubble to stem cells that are far from the vascular lumen. Because of this, most stem cell labeling using ultrasound has been targeted at adherence of stem cells to the vascular lumen or atherosclerotic plaque.

In a recent *in vitro* study, bifunctional, fluorescent and echogenic immunoliposomes (BF-ELIP) conjugated to anti-ICAM-1 antibodies for targeting to the atheroma were attached to bone marrow-derived CD34+ cells via anti-CD34 antibodies [101]. These BF-ELIP- labeled CD34+ bone marrow-derived stem cells were then incubated with freshly harvested porcine aortic tissue; ultrasound was then employed to enhance adhesion of the labeled cells to endothelium and enhance cell migration through the vessel wall rather than imaging [101].

In a similar vein, Toma *et al.* coated MSCs with cationic, gas-filled lipid microbubbles (mb-MSC), and used acoustic radiation generated by intravascular ultrasound at 1.7 MHz to encourage mb-MSCs delivered intra-aortically to adhere to the balloon-injured aorta in rabbits [102]. At 24 hours post-delivery, engrafted mb-MSCs remained localized to the luminal surface of the artery, but showed little migration beyond their original location on the luminal surface [102]. Recent refinements in this technique [103] are aimed at enhanced targeting of stem cells to specific sites of endothelial injury and additional mechanisms to encourage engraftment beyond the vessel itself.

Another approach is to use gold nanoparticles as acoustic reflectors. Nam *et al.* immobilized gold-labeled MSCs in a fibrin matrix and were able to perform longitudinal tracking [104]. Recently, internalization of polymer microbubbles by bone marrow-derived mesenchymal stem cells has been performed followed by *in vitro* ultrasound imaging [105]. The microbubbles, once internalized, remain acoustically active, and emit harmonics that are not exhibited by non-labeled stem cells. Unlike direct labeling with gold, if the cell died, one would anticipate that the microbubble shell integrity to degrade at which point detection of microbubble by ultrasound would cease as well.

Despite these advances, ultrasound tracking of stem cells is not widely employed as several significant hurdles must be overcome including: 1) the poor spatial resolution inherent with ultrasound imaging: 2) the intrinsic echogenicity of contrast agents preventing accurate cell quantification (agents cast acoustic shadows beneath the first unit of contrast encountered); 3) contrast agents can be diluted with each subsequent cell division; 4) agent stability is poor and higher stability may lead to uptake by phagocytic cells once cells die; 5) complicated acoustics result from uptake of the contrast agent into the cellular space; and 6) a limited field of view with the transthoracic, 2-dimensional-based technique thereby restricting access to many cardiac structures [82,106]. In 2012 using lessons learned from high intensity focused ultrasound and enhance gene therapy transfection with ultrasound, Ziadloo *et al.* used pulsed focused ultrasound (pFUS) in a non-destructive manner to enhance bone marrow stromal stem cell homing and retention after intravenous administration to tissue treated with pFUS [107]. Thus, ultrasound may be used in a variety of ways to enhance cellular therapeutic engraftment and to assess cardiac function after delivery even if not directly used to track cells.

#### CMR

Cardiovascular magnetic resonance (CMR) typically interrogates the distribution of water within a subject. The high spatial resolution of CMR, as well as its capabilities for generating images with three-dimensional (3D) anatomical detail and lack of ionizing radiation makes CMR attractive for clinical application.

#### Superparamagnetic- and paramagnetic-based cell tracking

CMR-based cellular tracking has been performed using paramagnetic and superparamagnetic contrast agents as well as non-proton-based contrast techniques. Detection of labeled cells is related to magnetic field strength, labeling efficiency, cell numbers, relaxivity and spatial resolution [83]. Paramagnetic agents, such as gadolinium chelates and dysprosium, act locally on nearby protons to cause them to relax faster thereby shortening T1 relaxation. Gadolinium-based contrast agents, which are approved for intravascular administration, are used extensively in combination with T1-weighted imaging to increase signal in the vessels for magnetic resonance angiography, dynamic perfusion assessment in the heart, and viability assessment in the heart in delayed contrast-enhanced imaging. However, for direct stem cell labeling, paramagnetic agents have poor sensitivity due to the decreased effect on extracellular water once the contrast agent is intracytoplasmic [108].

Iron oxide contrast agents are commonly categorized based on particle size as: 1) superparamagnetic iron oxide (SPIO), typically between 30–150 nm in diameter; 2) ultrasmall superparamagnetic iron oxide (USPIO), typically <30 nm in diameter; and 3) micron sized particles of iron oxide (MPIOs) typically >1000 nm in diameter. Superparamagnetic contrast agents create substantial disturbances in the local magnetic field, which leads to a rapid dephasing of protons. Gradient echo techniques that cannot compensate for these dephasing artifacts will show hypointensities in the vicinity of iron oxide particles irrespective of whether the nanoparticles are internalized into the cell or not [83]. Thus, the sensitivity for tracking cells labeled with iron oxide nanoparticles is much higher than paramagnetic agents. To maximize the sensitivity to the magnetic susceptibility effects of USPIO/SPIO/MPIOs, T2*-weighted sequences are typically used. However, image interpretation may become difficult due to other endogenous sources of magnetic susceptibility, including hemorrhage and tissue interfaces, which also can create hypointensities on T2*-weighted images. Nonetheless, SPIOs have been used in a number of non-cardiac clinical trials for cell tracking outside the United States [109-114].

**Figure 3:**
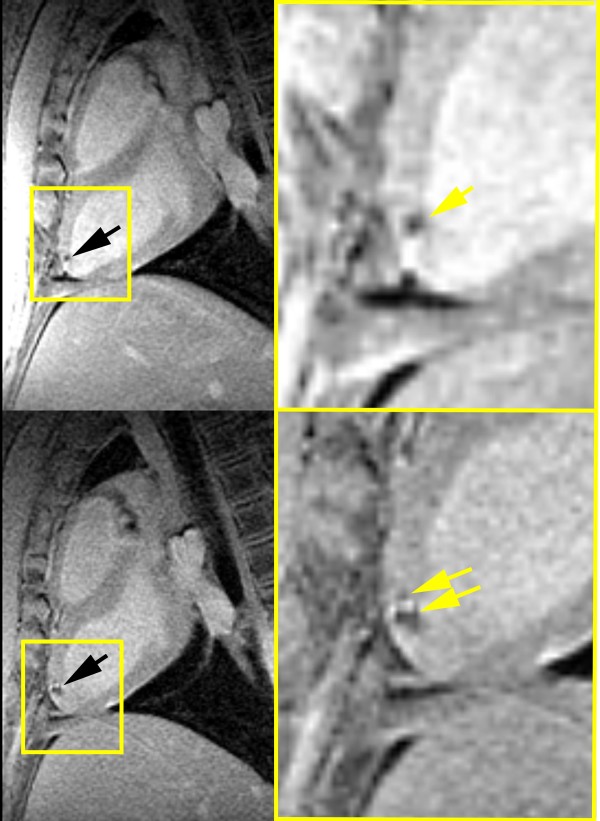
**Long**-**axis CMR showing hypointense lesions (arrows) caused by superparamagnetic iron oxide**-**labeled mesenchymal stem cells acquired within (top left) 24 h and (bottom left) 1 week of injection.** Insets demonstrate expansion of lesion over 1 week. Reprinted with permission from Kraitchman *et al.*[115].

Many preclinical studies have now been performed using iron oxide nanoparticles to help assess the optimal timing, stem cell type, dose, etc. In 2003, two studies heralded the use of iron oxide-labeled stem cells for cardiovascular cell delivery and tracking in large animals with CMR [115,116]. The first study by Kraitchman *et al.*[115] used a technique called “magnetofection” to label stem cells with ferumoxides, a clinically approved SPIO for liver imaging. In this study, the uptake of the ferumoxides in non-phagocytic, bone marrow-derived MSCs was enhanced by the addition of a transfection agent, poly-L-lysine. The concept of combining SPIOs with transfection agents to enable rapid cell labeling without species or cell specificity developed by Frank and Bulte [117]. In this first study, CMR tracking of the SPIO-labeled MSCs after transmyocardial injection was performed in a swine, reperfused myocardial infarction model (Figure 3). In this study, ~30% of the injections of SPIO-labeled stem cells injected under X-ray fluoroscopic guidance were not visible under CMR indicating that perceived successful injections did not occur. Extrapolating these results to clinical trials with non-labeled cells, one could anticipate that there may be a variable response in clinical trials due to intermittent failure to deliver the stem cells. Thus, without cellular labeling, it is impossible to assess whether the therapeutic failed because of failure of the stem cells to be delivered, failure of the therapeutic to be retained in the heart or failure of the therapy itself due to a poor choice of injection dose, cell type, or timing of injection.

**Figure 4:**
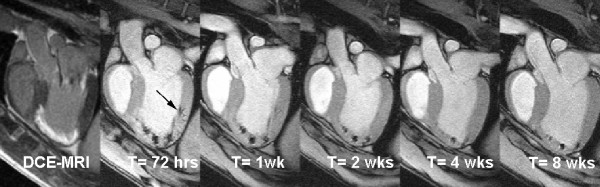
**Delayed contrast**-**enhanced (DCE) long**-**axis image (left) demonstrating infarcted myocardium (MI).** MR-labeled-MSC injections appear as hypointense areas on fast gradient echo images. Serial imaging at 72 hours, 1 week, 2 weeks, 4 weeks, and 8 weeks demonstrates the persistent of the MR-MSC injections. The volume of injections decreases over time. In addition, an injection placed in the normal myocardium (arrow) can no longer be detected at 4 weeks post-injection. Reprinted with permission from Soto *et al.*[120].

Hill *et al.* quickly followed with a similar study in swine using MPIO-labeled MSCs which were tracked up to three weeks after injection [116]. Building on this study, Dick *et al.* targeted MPIO-labeled MSCs to the infarct borders using a specialized active MR injection catheter and MR fluoroscopic imaging [118]. The advantage of the latter technique is that the success of the cell delivery could be immediately determined. Furthermore, using viability assessment with late gadolinium enhanced CMR [119], the cellular therapeutic could be specifically targeted to the peri-infarcted and infarcted tissue. In particular, Bulte and Kraitchman showed migration of stem cells in a reperfused dog infarction model over 8 weeks in the peri-infarction region [84]. SPIO-labeled MSCs were consistently removed when injected into normal myocardium whereas persistence of SPIO-labeled MSCs was noted in infarcted myocardium (Figure 4) [120].

Electromechanical mapping is another technique to target iron-labeled cellular therapy to viable myocardium and was first shown by Garot and colleagues for stem cell delivery in swine with infarcted myocardium [121]. In this study, injections in both normal and infarcted myocardium were all confirmed on CMR [121]. These results are in contrast to Kustermann and co-workers, who had difficulty differentiating the hypointensities created by USPIO-labeled cardiac progenitor cells from hypointensities due to the cryoablation or territory served by permanent coronary artery ligation to create damaged myocardium in mice. Presumably some of these issues were a result of the infarction model, small animal size, and small iron oxide particle size.

A variety of other investigators have studied iron oxide-labeled stem and progenitor cells with CMR [122-127]. In a novel approach, Weber *et al.*[122] used magnetic beads designed for cell sorting to select endothelial progenitor cells, i.e., CD34 magnetic beads. However, the long-term retention of these magnetic beads on cells *in vivo* has never been fully investigated. In another murine study, Tallheden *et al.* injected SPIO-labeled embryonic stem cells under direct visualization in the anterior left ventricle and demonstrated hypointensities consistent with the labeled cells [123]. Subsequently, two additional groups demonstrated long-term tracking of SPIO-labeled stem cells in infarcted hearts [124,125]. Stuckey *et al.* were able to track SPIO-labeled bone marrow-derived stromal cells up to 16 weeks post-infarction with CMR [124]. Ebert *et al.* not only tracked SPIO-labeled cardiac-differentiated mouse embryonic stem cells up to 28 days post-administration in a reperfused myocardial infarction, but also showed using CMR that declines in cardiac function were moderated by reductions in adverse remodeling [125]. Using MPIO-labeled amniotic fluid stem cells, Delo *et al.* were also able to demonstrate persistence of these cells up to 4 weeks after administration using CMR that was confirmed histologically [126]. Similarly, Chapon *et al.*[127] were able to demonstrate using CMR the persistence of hypointensities from rat bone marrow-derived stem cells labeled with a tat-peptide-USPIO nanoparticle [128] injected intramyocardially in both infarcted and sham operated rats at 6 weeks post-administration. A fluorescent label, FITC, in the tat-UPIO nanoparticle was used for histological validation. Despite using a 9.4 T CMR system, tracking of the hypointensities was more problematic, which the authors attributed to the smaller iron oxide nanoparticle used in this study.

Tracking of bone marrow-derived MSCs has been shown in a swine, reperfused infarction model by Hare and co-workers [129,130]. In the first study [129], the appearance of increased subendocardial myocardium in the infarct zone in close proximity to stem cell injections was shown on multi-detector CT, and the presence of contracting myocytes was confirmed using tagged CMR [131,132]. In a subsequent study [130], first pass contrast-enhanced CMR showed increased perfusion in the treated animals relative to controls prior to any functional benefit suggesting that MSCs may assist with angiogenesis and reduce apoptosis [130].

While these studies confirmed the presence of hypointensities on CMR corresponded to the exogenously labeled cells using histopathology, a number of studies have raised the concern that CMR hypointensities may reflect loss of the iron oxide nanoparticle from dead cells that either remains in the interstitial space or iron oxide nanoparticles that are taken up by phagocytic cells. In particular, Terrovitis *et al.* performed SPIO labeling of human and rat cardiac-derived stem cells (CDCs) that expressed beta-galactosidase and injected them intramyocardially in normal rats [133]. Persistence of hypointensities occurred for three weeks by CMR yet no CDCs were detected histologically. As expected, the area of the hypointensities decreased to a greater extent in the animals receiving xenogenic CDCs, presumably due to immunorejection, relative to the syngeneic CDCs whereas the hypointense area remained fairly constant from day 2 to day 21. Interestingly, the MR images in this study demonstrated a greater reduction in the hypointense area in the syngeneic animal compared to the xenogenic animal.

Schwaiger and colleagues performed a similar study in immunocompromised rats that received intramyocardial injections of iron oxide-labeled human endothelial progenitor cells (hEPCs) that were transfected with a PET reporter gene, the sodium iodide symporter (NIS) [134]. Both PET and CMR demonstrated the presence of the hEPCs at 24 hours post-injection. Hypointensities on CMR were still present at 72 hours and one week post-injection. On the other hand, PET imaging failed to detect any viable hEPCs at 1 week. Yet, immunohistochemistry at one week was unable to detect any of the exogenously hEPCs whereas staining for iron with Prussian Blue demonstrated co-localization of the iron with CD68+ cells, i.e., macrophages. While the authors note that PET imaging cannot detect less than 10,000 cells, the postmortem histology would suggest two things: 1. hEPCs did not survive even in an immune-compromised animal and 2. iron released from dying hEPCs is retained in macrophages that are still present at 1 week post-injection. In a related study, Li *et al.* performed serial imaging of SPIO-labeled human embryonic stem cells (hESCs) expressing a bioluminescence reporter gene that were injected into the hind limb of immunocompromised mice [135]. Undifferentiated ESCs are known for rapid proliferation and teratoma formation *in vivo*. As expected in this study, the development of teratoma resulted in an increased bioluminescence signal yet the CMR signal remained relatively constant. These reporter gene/CMR studies highlight two potential problems with direct labeling schemes: 1. that cell viability/proliferative capacity cannot be assessed and 2. that the label itself may become detached from the cell of interest. As such, direct labeling methods with SPIOs are best suited for targeting delivery and determining initial success rather than tracking cell viability. For long-term tracking, one must be cognizant that hypointensities may overestimate viable cells if free SPIOs or phagocytic cells, which scavenge the free SPIO, persist in the tissue. This issue is a problem in most cellular direct labeling methods.

**Figure 5:**
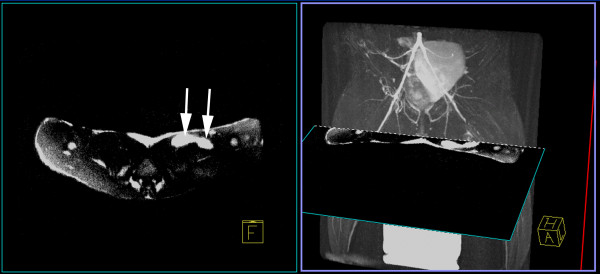
**Left: An axial positive contrast image using Inversion**-**Recovery with On**-**resonance water suppression (IRON) of SPIO**-**labeled stem cells injected in a rabbit thigh demonstrates two injection sites (arrows) as bright hyperintensities.** Right: A maximum intensity projection of a 3D T2-prepared MR angiogram shows the region of superficial femoral artery occlusion at 24 hours post-occlusion in a rabbit model of peripheral arterial disease can be registered with the IRON images to determine the location of stem cell injections relative to neovasculature. (Adapted with permission from Kraitchman and Bulte [108]).

Another issue with iron oxide nanoparticles is that the hypointensities created by the labeled cells compromise visualization of underlying tissue anatomy. Furthermore, other substances, such as air-tissue interfaces and metallic objects, e.g., stents, will create susceptibility artifacts that may mimic the hypointensities seen on T2*-weighted imaging of iron oxide-labeled cells. In an effort to overcome some of these issues, several techniques were developed based on a gradient dephasing technique by Seppenwoolde and co-workers [136] to passively track gadolinium-based markers, which cause local field inhomogeneities, in interventional devices. Using a twist on Seppenwoolde’s white marker technique and static dephasing theory of superparamagnetic particles explained by Bowen *et al.*[137], Mani *et al.* developed a slice-selective gradient echo technique for positive marker tracking of stem cells called GRASP [138-140]. However, these dephasing techniques are very sensitive to imaging parameters, such as slice thickness and echo time. Alternative positive contrast SPIO imaging techniques have been developed that employ spectrally selective excitation/suppression [141-143]. The spectrally-selective radiofrequency excitation and refocusing of off-resonant water frequencies proposed by Cunningham and colleagues [141] has been used *in vivo* to track embryonic stem cells implanted in the leg [135] and is best suited for spin echo techniques, which may limit applicability in the heart. An alternate off-resonant technique called inversion recovery with on-resonant water (IRON) saturates the water and fat peaks so that the off-resonant protons in close proximity to the SPIO-labeled stem cells are enhanced [142]. An added benefit to IRON imaging is that it can be used with either gradient echo or spin echo pulse sequences and can be applied across vendor platforms without any special pulse programming or post-processing of the images (Figure 5). Because these imaging techniques only preserve the susceptibility artifacts from the iron oxide-labeled cells, an additional anatomical reference image is needed. Alternatively Dahnke *et al.* have developed a post-processing method to determine the susceptibility gradient map from conventional gradient echo images to create positive contrast images of SPIO-labeled cell [144]. Thus, the standard anatomical image is obtained and no specialized pulse sequences are required. More recently, Garwood and coworkers have employed sweep imaging with Fourier transformation (SWIFT) for the detection of SPIO-labeled stem cells in the rat heart [145]. Using SWIFT, there is very little time between radiofrequency excitation and signal acquisition, which makes it well suited for imaging iron oxide labeling where the T2* relaxation time is very short. Interestingly, the magnitude image from SWIFT provides anatomical detail whereas the imaginary component provides a positive contrast image. However, the implementation of SWIFT is not available on commercial MR scanners nor is post-processing of the SWIFT images available currently. Another approach that has shown promise is to use ultrashort echo time (UTE) techniques available on clinical systems to acquire the T1 signature of iron oxide-labeled cells to create a positive contrast image [146,147].

At one time, when clinically approved SPIOs, i.e., ferumoxides and ferucarbotran, were available, translation of SPIO-labeling clinical cardiovascular stem cell trials showed promise because of ease of labeling and low toxicity of the label if only to confirm stem cell delivery. However, these SPIOs have ceased to be manufactured for economic reasons [148]. Interest in USPIO labeling has been renewed with several groups demonstrating an off-label use of an FDA-approved injectable ferumoxytol for the treatment of iron deficiency anemia in adults with chronic kidney disease (Feraheme, AMAG Pharmaceuticals) to label a variety of cells [149,150].

#### Non-proton labeling methods

**Figure 6:**
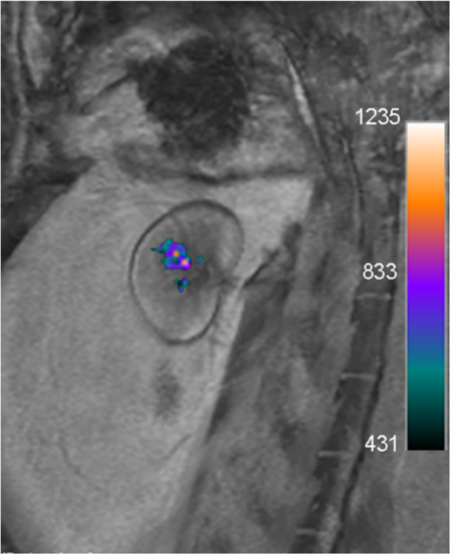
***In vivo *merged 19 F (color) and proton (grayscale) MRI acquired on a clinical 3T scanner of a rabbit transplanted with 10,000 perfluoropolyether (PFPE)**-**labeled islets under the kidney capsule demonstrates clear visualization of cell transplants.** The signal corresponds to 14,153 μg PFPE. Reprinted with permission from Barnett *et al.*[153].

One potential solution to the problems associated with direct cell labeling with iron oxide nanoparticles would be to use a label that is rapidly removed if the cell dies, similar to microbubbles, which are under development for ultrasound cell labeling. In 2003, Ahrens *et al.* exogenously labeled dendritic cells with perfluorocarbons and performed *in vivo* tracking after direct tissue or intravenous injection in mice using Fluorine (19 F) MRI [151]. Because there is very little native fluorine in the body apart from the teeth, one can be exquisitely sensitive to fluorine labeled cells with the creation of hot-spot MR images similar to SPECT and PET imaging. In 2007, Partlow *et al.* labeled human umbilical cord progenitor cells with two different perfluorocarbon nanoparticles and demonstrated the ability to detect as few as 2000 labeled cells at 11.7 T *in vivo* and 1 million cells *ex vivo* at 1.5 T [152]. These cells were labeled with the perfluorocarbons by simple incubation without a transfection agent. Presumably, should the cells die, the perfluorocarbon would be excreted via the lungs rather than being retained in tissue. Subsequently, Barnett *et al.* labeled pancreatic islet cells with these same two perfluorocarbons and performed the first *in vivo* studies showing fluorine CMR in a clinical 3T scanner with retention of the labeled cells in the renal capsule of rabbits (Figure 6) [153]. While the scanner must have multinuclear capabilities and transmit/receive coils to detect the fluorine signal, this study demonstrates the potential for translation to clinical studies. Further aiding possible clinical translation is the availability of clinically approved perfluorocarbon agents that are used for echocardiography and as blood substitutes.

Another strategy that was originally designed to enhance survival of allogeneic cell therapies is microencapsulation. In the 1980s, alginate microencapsulation was developed by Lim and Sun [154] as a method to immunoisolate islet cells for transplantation in Type I diabetes mellitus. Typically alginate microencapsulation is performed in a multi-layer approach, e.g., alginate-poly-L-lysine-alginate (APA), to enhance the porosity of the capsule while retaining strength and biocompatibility. Thus, the APA microencapsulation technique enhances survival of allogeneic or xenogenic cells by restricting the passage of large molecules, e.g. immunoglobulins, while simultaneously allowing small molecules, e.g., oxygen, nutrients, cytokines, and waste products, to exchange across the membrane. Initially, Barnett and coworkers recognized that the poly-L-lysine moiety could be used for iron oxide labeling of the microcapsule and exploited this concept for tracking islet cell transplantation [155,156]. More recently,this group has modified the formulation to incorporate perfluorocarbons for microcapsule tracking using fluorine MRI [157,158]. One immediate advantage of this technique is that higher concentrations of the labeling agent can be added to the microcapsule without affecting cell viability, thus enhancing sensitivity. Using positive contrast techniques [142], this group has also demonstrated that one may be able to monitor the integrity of the iron oxide-labeled cell capsules [155]. Arifin *et al.* have shown that gadolinium chelates may also be incorporated into the alginate microcapsules when linked to a gold nanoparticle, which are visualized as hyperintense signals on T1-weighted MR images at 2-days after delivery [159]. However, similar to direct labeling techniques, this method of tracking cannot directly report the viability of the cell.

#### Reporter gene imaging

MRI reporter gene-based labeling entails the transfection of genetic material with plasmids or viral vectors to induce the cell to produce a specific receptor, protein, or enzyme that usually can be detected by the introduction of a reporter probe. One of the advantages of reporter gene imaging is that only live cells will produce the reporter gene product. Furthermore, if the reporter gene is not constitutively expressed but rather only by a specific promoter, there is the potential of imaging stem cell fate once differentiation down a specific lineage has occurred.

One of the earliest examples of MR reporter genes enabled imaging of gene expression. The approach by Louie *et al.* was to use a paramagnetic contrast agent whose access to water was blocked until cleaved by an enzyme [160]. Thus, cells expressing the enzyme would create an active form of the paramagnetic contrast agent and show increased intensity on T1-weighted images.

Another approach to MR reporter gene imaging techniques was to transduce cells to overexpress a native protein, ferritin, which is responsible for iron storage within the cell. Thus, cells overexpressing ferritin will accumulate more iron within the cell leading to a signal amplification that can be several folds greater than with direct receptor binding to the cell. Campan *et al.* recently demonstrated overexpression of the human ferritin heavy chain (hFTH) as a MRI reporter gene for *in vivo* tracking of swine cardiac progenitor cells in the infarcted rat heart [161]. Lentiviral-transduced cardiospheres (CSCs) overexpressing hFTH were injected intramyocardially at the perimeter of the infarct [161]. Iron accumulation in the rat hearts was followed up to 4 weeks using a multiecho, T2* gradient echo sequence on a 1.5 T clinical scanner [161]. While there is some concern that increased iron accumulation in the cell may be detrimental, CSC differentiation down multiple lineages still occurred [161], which would suggest that stem cell pluripotency was retained despite genetic manipulation and iron uptake. An MR reporter gene approach that does not rely on MR superparamagnetic or paramagnetic compounds has been explored in the brain. Gilad *et al.* used overexpression of lysine-rich residues as an endogenous contrast agent with increased amide residues, which could exchange protons with water residues [162]. Using chemical exchange saturation transfer (CEST) imaging [163], transfected cells could be distinguished from non-transfected cells in the brain [162]. Because paired images with and without radiofrequency irradiation are required for CEST, cardiac motion may be extremely challenging for implementation of this reporter gene approach.

One of the primary potential advantages of MR reporter gene imaging is that the reporter gene should be passed to the daughter cells and, thus, issues associated with label dilution during cell proliferation/division is markedly reduced. On the other hand, one must make sure that genetic expression as well as uptake of the reporter probe does not affect cell viability and function. In addition, there are concerns about the long-term expression of the foreign genetic material. Fortunately, silencing of the reporter gene often occurs over time, which may reduce fears of delivering a genetically altered cells to young cardiovascular disease patients.

CMR has several remaining hurdles to overcome as it relates to stem cell therapy for cardiac applications. For instance, quantification of labeled cell populations is challenging with the direct labeling schemes as there is dilution of intracellular markers with every cell division and there is potential for accumulation of iron particles within phagocytic cells that may lead to the production of false signals. Further, there is low sensitivity relative to radionuclide imaging, a lack of compatible devices for real-time delivery using CMR, MR contraindications in many cardiac patients due to metallic implants, the high expense of the CMR equipment, a high degree of acoustic noise, and relatively poor physiological monitoring for the acute cardiac patient. However, the lack of exposure to ionizing radiation to the patient, operator, and stem cells themselves in addition to the ability to determine myocardial viability, function, and perfusion in a regional manner have resulted in more frequent use of CMR to assess patients after stem cell delivery in cardiovascular clinical trials [5-7,9,74,75,78,79,164,165].

#### X-ray

X-ray based imaging modalities include computed tomography (CT) imaging and X-ray fluoroscopy. The high spatial resolution and real-time interactivity are highly desirable attributes of these X-ray-based imaging modalities for clinical assessment and treatment of the heart. However, concerns about ionizing radiation dose and the limited ability to directly visualize soft tissue, such as the myocardium, are major drawbacks. Furthermore, most X-ray contrast agents are not amenable for direct cell labeling due to their high toxicity at relatively low doses.

Recently, Ricel *et al.* have develop a gold nanoparticles coated with poly-L-lysine for direct stem cell labeling [166]. To enhance biocompatibility, gold nanoparticles are typically coated with substances, such as polyethylene glycol (PEG), which in turn inhibits direct uptake of the nanoparticle by stem cells. Unlike MRI labeling compounds, gold nanoparticles have been shown to be actively expelled via exocytosis by cells [167]. Thus, loss of a gold label may occur even without cell division.

**Figure 7:**
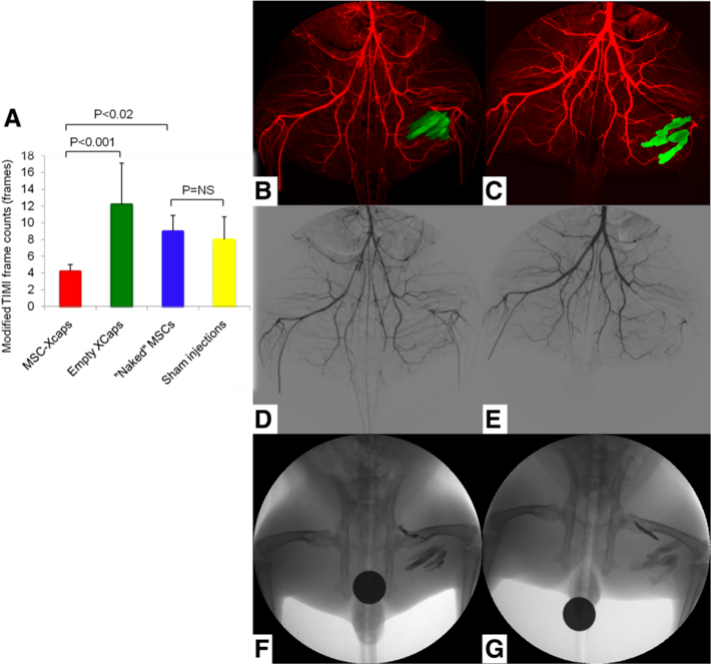
**Barium sulfate-labeled microcapsules for X-ray cell tracking (Xcaps) in peripheral arterial disease. (A)** A bar graph of the average modified Thrombolysis In Myocardial Infarction *(*TIMI) frame count, as a measure of collateral vessel development, in the MSC-Xcaps, empty microcapsules, unencapsulated MSCs, and sham injection treated animals demonstrating a significant improvement in distal filling only in the peripheral arterial disease (PAD) rabbits that received microencapsulated cells (*P < 0.001 empty microcapsules vs. MSC-Xcaps; P = NS naked MSCs vs. sham injections). B-G: Representative digital subtraction angiogram (DSA, red) obtained during peak contrast opacification performed at two weeks post injection of encapsulated MSCs-Xcaps **(B)** and empty microcapsules **(C)** with an overlay of microcapsules injections (green) obtained from mask image of DSA. The small collateral vessels are somewhat obscured by the Xcap radiopacity. However, the increased collateralization can be appreciated in the MSC-Xcap-treated animal DSA **(D)** relative to the Xcap-treated animal **(E)** Native mask digital radiographs demonstrating the location of the MSC-Xcaps **(F)** and empty Xcaps **(G)** in the same animals. There was no statistically significant difference in vessel diameter between treatment groups. Reprinted with permission from Kedziorek *et al.*[171].

**Figure 8:**
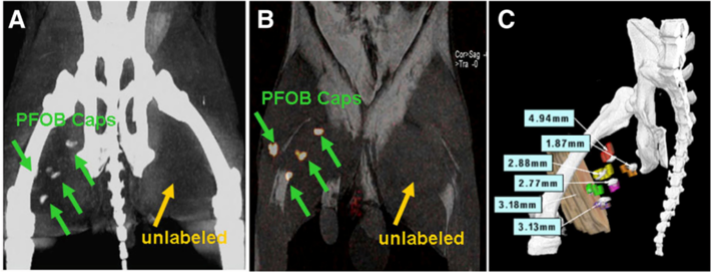
**Perfluorocarbon-labeled microcapsules for X-ray visible cell tracking by CT. (A)** Cone beam CT acquired on a flat-panel X-ray angiographic system (Axiom Artis, Siemens AG, Forchheim, Germany) demonstrating the detection of four perfluorooctylbromide (PFOB) injection sites in a rabbit medial thigh, while unlabeled capsules in the left thigh are not detectable. **(B)** 19 F MRI of the same rabbit showing one-to-one correspondence to the injection location on cone beam CT. **(C)** Co-registering of threshold cone beam CT image of a rabbit with 6 PFOB Caps injection sites (gray) and postmortem 3D rendering volume of each injection sites (color) demonstrating the location of opacities on cone beam CT image is representative of PFOB Caps injections. Registration error for each injection site from a representative rabbit is shown. Reprinted with permission from Fu *et al.*[172].

The development of MR-labeled alginate microcapsules has also been translated to X-ray-visible microcapsules. As with MRI, this allows the use of high concentrations of X-ray-visible contrast agents in the microcapsules without cellular toxicity for tracking using conventional X-ray fluoroscopic and computed tomography (CT) [156,157,168,169]. Impregnation of APA microencapsulation with barium or bismuth sulfate allowed *in vivo* tracking of microcapsules in mice and rabbits using conventional X-ray fluoroscopy [156,168]. These bariumsulfate-labeled microcapsules were also used to confirm delivery and retention of allogeneic MSCs in a rabbit model of peripheral arterial disease (Figure 7) [169-171]. Single microcapsules could be seen *in vitro* using cone beam CT, although, in practice, several thousand microcapsules would be expected as a minimum to achieve a therapeutic effect. Another formulation using perfluoroctylbromide (PFOB) enables capsule tracking with X-ray, MRI, and ultrasound and may be useful for allowing delivery using X-ray fluoroscopic or ultrasound techniques while reducing radiation dose by using MR or ultrasound imaging for follow-up (Figure 8) [157].

**Figure 9:**
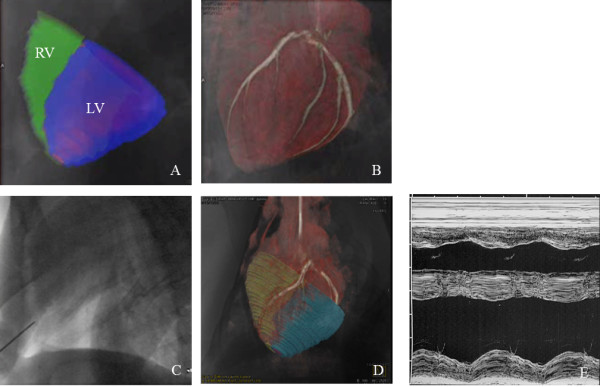
**X-ray fused with MRI (XFM) of X-ray-visible microcapsules to the heart. (A)** Segmented cine CMR showing epicardial contours (green-RV; blue-LV) overlaid on live X-ray fluoroscopic image. **(B)** Coronary vasculature from c-arm CT overlaid on live X-ray fluoroscopic image. **(C)** Live X-ray fluoroscopy demonstrating radiopacity of needle used for pericardial puncture and the lack of ability to visualize the myocardium or coronary vasculature without XFM. **(D)** Live X-ray fluoroscopy image overlaid on segmented whole heart CMR and c-arm CT volumes showing pericardial puncture. **(E)** An M-mode echocardiogram at seven days post-injection demonstrating normal cardiac function and no abnormalities to the pericardium. Reprinted with permission from Azene *et al.*[173].

One hurdle for application of microencapsulation techniques in the heart is the large size of the microcapsules, which is ~300-500 μm. Thus, this labeling method is not amenable to intravascular or transmyocardial delivery due to concern about vascular occlusion or induction of conduction abnormalities. As such, these techniques may be better suited for treatment outside the heart. Moreover, since the cells are trapped within the microcapsule, direct incorporation into the myocardium is unlikely. In 2011, Azene *et al.* demonstrated that an alternate delivery site for X-ray-visible microcapsules may be the pericardial space [173]. Using myocardial borders derived from a navigator- and cardiac-gated whole heart CMR at 1.5T fused with real-time X-ray fluoroscopy, barium sulfate impregnated microcapsules were delivered to the pericardial space in swine on a clinical angiographic system (Figure 9) [173]. These studies build upon X-ray fused with MRI (XFM) techniques [174,175] developed in preclinical applications [176] for ultimate translation to pediatric and adult cardiac interventions [177,178].

#### Summary

In the past 10 years, there have been a large number of preclinical studies and clinical trials that have used CMR to delivery, track, or determine the efficacy of stem cell therapy in the heart. While X-ray cell labeling techniques are not as mature at present for translation to clinical trials, the ability to fuse multiple image modalities, such as radionuclide imaging, CT, or MRI, with X-ray fluoroscopic imaging offers the ability to obtain the optimal interact interface for cell delivery with anatomical, functional, and metabolic information to better guide stem cell administration to the heart and peripheral vasculature.

## Endnote

^a^http://www.who.int/mediacentre/factsheets/fs317/en/index.html.

## Competing interests

The authors declare that they have no competing interests.

## Authors’ contributions

NA, YF, JM, and DLK drafted, read, and approved the final manuscript.
